# Patient and Caregiver Experiences of Navigating the “Seesaw” of Hospice Live Discharge

**DOI:** 10.1093/geroni/igaf122.2009

**Published:** 2025-12-31

**Authors:** Cara Wallace, Stephanie P Wladkowski, Todd Becker, Joseph Trinitas, Ruaa Al Juboori, Leslie Hinyard, Verna Hendricks-Ferguson

**Affiliations:** Saint Louis University, Saint Louis, Missouri, United States; Bowling Green State University, Bowling Green, Ohio, United States; WashU Medicine, St. Louis, Missouri, United States; Saint Louis University, St. Louis, Missouri, United States; University of Mississippi, Oxford, Mississippi, United States; Saint Louis University School of Medicine, St. Louis, Missouri, United States; Trudy Busch Valentine School of Nursing, St. Louis, Missouri, United States

## Abstract

The receipt of quality hospice care may prompt changes in patient eligibility under Medicare’s prognostic requirement of 6 months or less. In 2022, 224,900 hospice beneficiaries across the United States were discharged alive from hospice care, with 6.1% discharged due to “no longer being terminally ill.” This critical care transition is associated with losing important hospice-based supports and questions if to re-enroll back onto hospice care. Previous research is limited on how patients and caregivers navigate countervailing positive and negative reactions that shape this phenomenon. The current study aimed to address this gap by exploring the experience of service coordination and potential impacts to quality-of-life post-discharge. With a purposive sample of 32 patient-caregiver dyads, in-depth, semi-structured interviews were analyzed via thematic analysis. Results were characterized by navigating a process of ups and downs with varying questions about whether the patient was maintaining eligibility, akin to a seesaw. Specifically, caregivers described an uncertainty regarding how hospice patients managed to “get better” to warrant discharge, when their needs for assistance remained the same. Other caregivers cast doubt on if discharge was necessary or even appropriate. Whereas nearly half of participants referred to live discharge as incongruent with their goals for care, the experiences associated with navigating live discharge (both positive and negative) led others to question if they would want the patient to re-enroll in hospice in the future. These results suggest a need to develop and examine robust services to support patient and caregiver transitions from hospice care.

